# Application of an Externally Developed Algorithm to Identify Research Cases and Controls from EHR Data: Trials and Triumphs

**DOI:** 10.1055/a-2524-5216

**Published:** 2025-03-26

**Authors:** Nelly Estefanie Garduno-Rapp, Simone Herzberg, Henry H. Ong, Cindy Kao, Christoph U. Lehmann, Srushti Gangireddy, Nitin B Jain, Ayush Giri

**Affiliations:** 1 Clinical Informatics Center, University of Texas Southwestern Medical Center, Dallas, Texas, United States; 2 Division of Epidemiology, Department of Medicine, Vanderbilt University Medical Center, Nashville, Tennessee, United States; 3 Medical Scientist Training Program, Vanderbilt University School of Medicine, Nashville, Tennessee, United States; 4 Center for Precision Medicine, Department of Biomedical Informatics, Vanderbilt University Medical Center, Nashville, Tennessee, United States; 5 Department of Physical Medicine and Rehabilitation, University of Michigan, Ann Arbor, Michigan, United States; 6 Division of Quantitative and Clinical Sciences, Department of Obstetrics and Gynecology, Vanderbilt University Medical Center, Nashville, Tennessee, United States

**Keywords:** phenotypic algorithms, data validation, clinical research informatics

## Abstract

**Background:**

The use of electronic health records (EHRs) in research demands robust and interoperable systems. By linking biorepositories to EHR algorithms, researchers can efficiently identify cases and controls for large observational studies (e.g., genome-wide association studies). This is critical for ensuring efficient and cost-effective research. However, the lack of standardized metadata and algorithms across different EHRs complicates their sharing and application. Our study presents an example of a successful implementation and validation process.

**Objectives:**

This study aimed to implement and validate a rule-based algorithm from a tertiary medical center in Tennessee to classify cases and controls from a research study on rotator cuff tear (RCT) nested within a tertiary medical center in North Texas and to assess the algorithm's performance.

**Methods:**

We applied a phenotypic algorithm (designed and validated in a tertiary medical center in Tennessee) using EHR data from 492 patients enrolled in a case-control study recruited from a tertiary medical center in North Texas. The algorithm leveraged the international classification of diseases and current procedural terminology codes to identify case and control status for degenerative RCT. A manual review was conducted to compare the algorithm's classification with a previously recorded gold standard documented by clinical researchers.

**Results:**

Initially the algorithm identified 398 (80.9%) patients correctly as cases or controls. After fine-tuning and correcting errors in our gold standard dataset, we calculated a sensitivity of 0.94 and a specificity of 0.76. The implementation of the algorithm presented challenges due to the variability in coding practices between medical centers. To enhance performance, we refined the algorithm's data dictionary by incorporating additional codes. The process highlighted the need for meticulous code verification and standardization in multi-center studies.

**Conclusion:**

Sharing case-control algorithms boosts EHR research. Our rule-based algorithm improved multi-site patient identification and revealed 12 data entry errors, helping validate our results.

## Background and Significance

As the use of electronic health records (EHRs) for large-scale research is increasing, 1 there is a pressing need to develop robust infrastructures and innovative research tools to provide syntactic and semantic interoperability among health systems and organizations. 2 3 To achieve this concept, researchers must overcome the lack of harmonization of national and institution-specific terminologies, formats, and structures into standardized formats such as the observational medical outcomes partnership and common data model. 2 4 5 6 Such advancements could transform EHRs into powerful research tools and ultimately contribute to improved patient outcomes. A critical aspect of this transformation involves the development of harmonized models, techniques, tools, and algorithms that can be applied to large datasets across multiple health systems. 5 7 8 One prominent type of research that leverages large-scale datasets and often involves data collected from multiple sites are genome-wide association studies (GWAS), 9 which are increasingly prevalent and identify genetic variants that predispose individuals to complex disorders (association between genotype and phenotype). 10 These studies hold great promise for advancing our understanding and treatment of various diseases such as degenerative rotator cuff tear (DCT), with the caveat that data from EHRs, originally collected for patient care rather than research, are curated in a principled manner. 11 12

A fundamental component of the success of population studies, including GWAS, is the correct classification of cases and controls. 13 14 While various cohort discovery tools, such as i2b2 (informatics for integrating biology at the bedside), TriNetX, and OHDSI/ATLAS (observational health data sciences and informatics), quickly facilitate the identification of potential research participants, these tools are most effective for direct, single-step queries. 15 16 These platforms have fixed structures for how the data are stored and organized, which could limit the flexibility in how data are queried or analyzed. Thus, they fall short when handling complex clinical scenarios and meeting specific criteria that require multi-step temporal logic to answer research questions. 17

Our study addresses this gap by implementing and validating an external rule-based algorithm, leveraging current procedural terminology (CPT) and international classification of diseases (ICD) coding. Algorithms based on CPT and ICD codes offer a more effective approach, due to their flexibility to tailor data and rules to classify cases and controls in a more precise way. This allows for more accurate categorization in complex scenarios, overcoming the limitations of traditional cohort discovery tools. 18 19 20

Nonetheless, research has shown that structured algorithms must be clear and well-defined to avoid poor interpretation. For instance, asking for “patients that are 40 years of age or older” does not indicate at what point in the disease course the patient should be at least 40. 21 22

The algorithm used in this study was developed using a unique combination of CPT and ICD codes and it involved consideration of frequency and temporality associated with other codes. It was designed and internally validated at Vanderbilt University Medical Center (VUMC) from a de-identified clinical records database. The database supports queries of structured clinical information such as diagnostic codes, CPT codes, medications, laboratory data, allergies, and demographics, and unstructured clinical information including medical reports, radiology notes, and surgical notes. More details of the VUMC algorithm are described elsewhere. 23

Briefly, UT Southwestern Medical Center (UTSW) and VUMC are both tertiary medical centers with diverse populations in the southern United States. This makes our study particularly valuable by demonstrating the algorithm's performance across different EHR instances.

In this work, we provide a comprehensive account of the algorithm's implementation and validation processes. We demonstrate how applying this external algorithm contributed to greater consistency and reliability in our case and control classifications within the gold-standard dataset.

### Hypothesis

We hypothesized that the algorithm developed at VUMC would initially underperform and miss cases and controls from our gold standard dataset at UTSW, and that targeted improvements could enhance its performance and usability across other tertiary medical centers.

### Objective

To implement and validate a rule-based algorithm designed at VUMC to classify RCT cases and controls in a tertiary care medical center at UTSW and to evaluate the algorithm performance.

## Methods

### Study Population

Patients older than 40 years of age with a shoulder magnetic resonance image (MRI) met the eligibility criteria for enrollment in an actively recruiting observational, case-control study for a GWAS at UTSW, which served as the gold-standard case-control classifications. Cases in this study were determined based on the presence of a shoulder MRI with evidence of an atraumatic RCT as documented in the patient's medical chart. Controls were patients with a shoulder MRI indicating a condition other than RCT, such as adhesive capsulitis, osteoarthritis, or shoulder instability. Trained research personnel recorded patient information and classification as a case or as the control in a web-based data collection tool (REDCap) as the gold standard for this study. 24

### Processing the Gold Standard Dataset

Initially, we downloaded a de-identified dataset from REDCap, which included the current case or control classifications for 492 participants (405 cases and 87 controls) who were enrolled from 2021 to 2023. This dataset was maintained as our gold standard for subsequent analysis. Although this dataset lacked personal identifiers, each entry was associated with a unique, study-specific identifier that allowed us to align records accurately across datasets.

### Applying the Algorithm Developed at VUMC to the UTSW EHR Databases

Next, we applied the VUMC algorithm to all 492 participants in our epic databases, specifically: Caboodle and Clarity. The algorithm employed specific combinations of 18 CPT codes, 13 ICD-9-CM codes, and 39 ICD-10-CM codes. This ensured the precise identification of participants with RCTs while distinguishing them from those with other shoulder conditions, such as adhesive capsulitis, glenohumeral osteoarthritis (GHOA), or scapular dyskinesis.

Additionally, the algorithm had frequency and temporality requirements: 1) To ensure accuracy, the codes needed to be mentioned more than once at separate time points in the medical record, and 2) codes had to satisfy temporal relationship requirements with other codes. For example, to become a case, a patient had to have a CPT code for a shoulder MRI followed by an ICD code for RCT diagnosis within 1 year after the CPT code. Tables 1 and 2 display the full algorithm criteria. Table 3 displays our full data dictionary.

**Table 1:** Algorithm criteria for cases

	Case definition	Description	Criteria and Boolean logic
1	Specific surgical Inclusion	The Dx date is determined by the earliest date associated with a specific surgical procedure	a. rct_cpt_surg_spec_include
2	Non-specific surgical/ICD inclusion	The Dx date is the earliest date associated with non-specific surgical procedures or ICD codes, with additional criteria for diagnosis within a year	a. rct_cpt_surg_nonspec_include b. AND ( rct_icd9_diag_include OR rct_icd10_diag_include within 1 year after)
3	Imaging and diagnosis	The Dx date is based on imaging CPT/ICD codes, with diagnosis codes within a year and exclusion criteria applied afterward	a. (rct_cpt_image_include OR RCT_icd9_image_include) b. AND (rct_icd9_diag_include OR rct_icd10_diag_include within 1 year after) c. NOT (rct_icd9_exclusions OR rct_icd10_exclusions after CPT/ICD image include codes)
4a	Multiple ICD inclusions (3 visits)	The Dx date is determined by the third unique ICD code, ensuring there are 3 visits with the relevant diagnosis without exclusion codes	a. ≥3 unique visits with mentions of rct_icd9_diag_include OR rct_icd10_diag_include b. NOT rct_icd9_exclusions OR rct_icd10_exclusions (After ICD inclusion codes)
4b	Multiple ICD inclusions (4 visits)	The Dx date is set by the fourth unique ICD code, ensuring there are at least four mentions of the relevant diagnosis without exclusion codes	a. ≥4 mentions of rct_icd9_diag_include OR ≥ 4 mentions of rct_icd10_diag_include b. NOT rct_icd9_exclusions OR rct_icd10_exclusions (After ICD diag include)

Abbreviations: CPT, current procedural terminology; Dx, diagnosis; ICD, international classification of diseases.

**Table 2:** Algorithm criteria for controls

	Control definition	Description	Criteria and Boolean logic
1	Any non-case	Any non-case patients from the CPT/ICD list	a. NOT case status from CPT/ICD codes
2	CPT/ICD only with Imaging code confirmation for in-tact rotator cuff	All patients with CPT codes for imaging. All patients with ICD-9 codes for imaging. All patients with ICD-10 codes for imaging and exclusion criteria applied afterward. If the patient meets the criteria for being a case, they are excluded from the control group	a. (rct_cpt_image_include OR RCT_icd9_image_include OR RCT_icd10_image_include) b. NOT case status from CPT/ICD codes c. NOT (rct_cpt_surg_spec_include OR rct_cpt_surg_nonspec_include OR RCT_icd9_diag_include OR RCT_icd10_diag_include)

Abbreviations: CPT, current procedural terminology; ICD, international classification of diseases.

**Table 3:** Data dictionary list

Variable name	Type	Code	Code name/description	UTSW only	VUMC only	Shared in both
rct_cpt_surg_spec_include	ICD9CM	83.63	Rotator cuff repair	No	No	Yes
RCT_icd9_diag_include	ICD9CM	727.61	Complete rupture of rotator cuff	No	No	Yes
RCT_icd9_diag_include	ICD9CM	726.13	Partial tear of rotator cuff	No	No	Yes
RCT_icd9_diag_include	ICD9CM	83.63	Rotator cuff repair	No	No	Yes
RCT_icd9_nontraum	ICD9CM	727.6	Rupture of tendon nontraumatic	No	No	Yes
RCT_icd9_nontraum	ICD9CM	727.6	Nontraumatic rupture of unspecified tendon	No	No	Yes
RCT_icd9_exclusions	ICD9CM	840.3	Infraspinatus (muscle; tendon) sprain	No	No	Yes
RCT_icd9_exclusions	ICD9CM	840.4	Rotator cuff (capsule) sprain	No	No	Yes
RCT_icd9_exclusions	ICD9CM	840.5	Subscapularis (muscle) sprain	No	No	Yes
RCT_icd9_exclusions	ICD9CM	840.6	Supraspinatus (muscle; tendon) sprain	No	No	Yes
RCT_icd9_image_include	ICD9CM	88.94	Magnetic resonance imaging of musculoskeletal	No	No	Yes
RCT_icd9_image_include	ICD9CM	88.32	Contrast arthrogram	No	No	Yes
RCT_icd9_image_include	ICD9CM	88.7	Diagnostic ultrasound	No	No	Yes
RCT_icd10_diag_include	ICD10CM	M75.120	Complete rotator cuff tear or rupture of unspecified shoulder, not specified as traumatic	No	No	Yes
RCT_icd10_diag_include	ICD10CM	M75.121	Complete rotator cuff tear or rupture of right shoulder, not specified as traumatic	No	No	Yes
RCT_icd10_diag_include	ICD10CM	M75.122	Complete rotator cuff tear or rupture of left shoulder, not specified as traumatic	No	No	Yes
RCT_icd10_diag_include	ICD10CM	M75.110	Incomplete rotator cuff tear or rupture of unspecified shoulder, not specified as traumatic	No	No	Yes
RCT_icd10_diag_include	ICD10CM	M75.111	Incomplete rotator cuff tear or rupture of right shoulder, not specified as traumatic	No	No	Yes
RCT_icd10_diag_include	ICD10CM	M75.112	Incomplete rotator cuff tear or rupture of left shoulder, not specified as traumatic	No	No	Yes
RCT_icd10_diag_include	ICD10CM	M75.100	Unspecified rotator cuff tear or rupture of unspecified shoulder, not specified as traumatic	No	No	Yes
RCT_icd10_diag_include	ICD10CM	M75.101	Unspecified rotator cuff tear or rupture of right shoulder, not specified as traumatic	No	No	Yes
RCT_icd10_diag_include	ICD10CM	M75.102	Unspecified rotator cuff tear or rupture of left shoulder, not specified as traumatic	No	No	Yes
rct_icd10_exclude	ICD10CM	S46.011A	Strain of muscle(s) and tendon(s) of the rotator cuff of right shoulder, initial encounter	No	No	Yes
rct_icd10_exclude	ICD10CM	S46.011D	Strain of muscle(s) and tendon(s) of the rotator cuff of right shoulder, subsequent encounter	No	No	Yes
rct_icd10_exclude	ICD10CM	S46.011S	Strain of muscle(s) and tendon(s) of the rotator cuff of right shoulder, sequela	No	No	Yes
rct_icd10_exclude	ICD10CM	S46.012A	Strain of muscle(s) and tendon(s) of the rotator cuff of left shoulder, initial encounter	No	No	Yes
rct_icd10_exclude	ICD10CM	S46.012D	Strain of muscle(s) and tendon(s) of the rotator cuff of left shoulder, sequential encounter	No	No	Yes
rct_icd10_exclude	ICD10CM	S46.012S	Strain of muscle(s) and tendon(s) of the rotator cuff of left shoulder, sequela	No	No	Yes
rct_icd10_exclude	ICD10CM	S46.011A	Strain of muscle(s) and tendon(s) of the rotator cuff of unspecified shoulder, initial encounter	No	No	Yes
rct_icd10_exclude	ICD10CM	S46.011D	Strain of muscle(s) and tendon(s) of the rotator cuff of unspecified shoulder, subsequent encounter	No	No	Yes
rct_icd10_exclude	ICD10CM	S46.011S	Strain of muscle(s) and tendon(s) of the rotator cuff of unspecified shoulder, sequela	No	No	Yes
rct_icd10_exclude	ICD10CM	S46.021A	Laceration of muscle(s) and tendon(s) of the rotator cuff of the right shoulder, initial encounter	No	No	Yes
rct_icd10_exclude	ICD10CM	S46.021D	Laceration of muscle(s) and tendon(s) of the rotator cuff of the right shoulder, sequential encounter	No	No	Yes
rct_icd10_exclude	ICD10CM	S46.021S	Laceration of muscle(s) and tendon(s) of the rotator cuff of the right shoulder, sequela	No	No	Yes
rct_icd10_exclude	ICD10CM	S46.022A	Laceration of muscle(s) and tendon(s) of the rotator cuff of the left shoulder, initial encounter	No	No	Yes
rct_icd10_exclude	ICD10CM	S46.022D	Laceration of muscle(s) and tendon(s) of the rotator cuff of the left shoulder, Sequential encounter	No	No	Yes
rct_icd10_exclude	ICD10CM	S46.022S	Laceration of muscle(s) and tendon(s) of the rotator cuff of the left shoulder, sequela	No	No	Yes
rct_icd10_exclude	ICD10CM	S46.029A	Laceration of muscle(s) and tendon(s) of the rotator cuff of unspecified shoulder, initial encounter	No	No	Yes
rct_icd10_exclude	ICD10CM	S46.029D	Laceration of muscle(s) and tendon(s) of the rotator cuff of unspecified shoulder, Sequential encounter	No	No	Yes
rct_icd10_exclude	ICD10CM	S46.029S	Laceration of muscle(s) and tendon(s) of the rotator cuff of unspecified shoulder, sequela	No	No	Yes
rct_icd10_exclude	ICD10CM	S43.421A	Sprain of muscle(s) and tendon(s) of the rotator cuff of right shoulder, initial encounter	No	No	Yes
rct_icd10_exclude	ICD10CM	S43.421D	Sprain of muscle(s) and tendon(s) of the rotator cuff of right shoulder, sequential encounter	No	No	Yes
rct_icd10_exclude	ICD10CM	S43.421S	Sprain of muscle(s) and tendon(s) of the rotator cuff of right shoulder, sequela	No	No	Yes
rct_icd10_exclude	ICD10CM	S43.422A	Sprain of muscle(s) and tendon(s) of the rotator cuff of left shoulder, initial encounter	No	No	Yes
rct_icd10_exclude	ICD10CM	S43.422D	Sprain of muscle(s) and tendon(s) of the rotator cuff of left shoulder, sequential encounter	No	No	Yes
rct_icd10_exclude	ICD10CM	S43.422S	Sprain of muscle(s) and tendon(s) of the rotator cuff of left shoulder, sequela	No	No	Yes
rct_icd10_exclude	ICD10CM	S43.429A	Sprain of muscle(s) and tendon(s) of the rotator cuff of unspecified shoulder, initial encounter	No	No	Yes
rct_icd10_exclude	ICD10CM	S43.429D	Sprain of muscle(s) and tendon(s) of the rotator cuff of unspecified shoulder, sequential encounter	No	No	Yes
rct_icd10_exclude	ICD10CM	S43.429S	Sprain of muscle(s) and tendon(s) of the rotator cuff of unspecified shoulder, sequela	No	No	Yes
rct_icd10_exclude	ICD10CM	M12.511	Traumatic arthropathy, right shoulder	No	No	Yes
rct_icd10_exclude	ICD10CM	M12.512	Traumatic arthropathy, left shoulder	No	No	Yes
rct_icd10_exclude	ICD10CM	M12.519	Traumatic arthropathy, unspecified shoulder	No	No	Yes
rct_cpt_surg_spec_include	CPT	23412	Repair of ruptured musculotendinous cuff (e.g., rotator cuff) open; chronic	No	No	Yes
rct_cpt_surg_spec_include	CPT	23420	Reconstruction of complete shoulder (rotator) cuff avulsion; chronic	No	No	Yes
rct_cpt_surg_spec_include	CPT	29827	Arthroscopy, shoulder, surgical; with rotator cuff repair	No	No	Yes
rct_cpt_surg_nonspec_include	CPT	80.21	Arthroscopy, shoulder	Yes	No	No
rct_cpt_surg_nonspec_include	CPT	29826	Arthroscopy, shoulder, surgical; decompression of subacromial space with partial acromioplasty, with coracoacromial ligament (i.e., arch) release, when performed	No	No	Yes
rct_cpt_surg_nonspec_include	CPT	29805	Arthroscopy, shoulder, diagnostic, with or without synovial biopsy (separate procedure)	Yes	No	No
rct_cpt_surg_nonspec_include	CPT	29822	Arthroscopy, shoulder, surgical; debridement, limited	No	No	Yes
rct_cpt_surg_nonspec_include	CPT	29823	Arthroscopy, shoulder, surgical; debridement, extensive	No	No	Yes
rct_cpt_surg_nonspec_include	CPT	01610	Anesthesia for all procedures on nerves, muscles, tendons, fascia, and bursae of the shoulder and axilla	Yes	No	No
rct_cpt_surg_nonspec_include	CPT	01622	Anesthesia for diagnostic arthroscopic procedures of shoulder joint	Yes	No	No
rct_cpt_surg_nonspec_include	CPT	01630	Anesthesia for open or surgical arthroscopic procedures on humeral head and neck, sternoclavicular joint, acromioclavicular joint, and shoulder joint; not otherwise specified	Yes	No	No
rct_cpt_surg_nonspec_include	CPT	01638	Anesthesia for open or surgical arthroscopic procedures on humeral head and neck, sternoclavicular joint, acromioclavicular joint, and shoulder joint; total shoulder replacement	Yes	No	No
rct_cpt_surg_nonspec_include	CPT	01710	Anesthesia for procedures on nerves, muscles, tendons, fascia, and bursae of upper arm and elbow; not otherwise specified	Yes	No	No
rct_cpt_image_include	CPT	23350	Injection procedure for shoulder arthrography or enhanced CT/MRI shoulder arthrography	No	No	Yes
rct_cpt_image_include	CPT	73221	MRI of shoulder, elbow, wrist, or clavicle w/o contrast	No	No	Yes
rct_cpt_image_include	CPT	73223	MRI of shoulder, elbow, wrist, or clavicle w/o contrast	No	No	Yes
rct_cpt_image_include	CPT	73218	MRI upper extremity w/o contrast	No	No	Yes
rct_cpt_image_include	CPT	73220	MRI of upper extremity w/o contrast involvement	No	No	Yes
rct_cpt_image_include	CPT	76140	CT/MR/MRA outside study	Yes	No	No
rct_cpt_surg_nonspec_include	CPT	23410	Repair of ruptured musculotendinous cuff (e.g., rotator cuff) open; chronic	No	Yes	No
rct_cpt_surg_nonspec_include	CPT	23397	Under repair, revision, and/or reconstruction procedures on the shoulder	No	Yes	No
rct_cpt_surg_nonspec_include	CPT	29901	Under endoscopy/arthroscopy procedures on the musculoskeletal system	No	Yes	No
rct_cpt_surg_exclude	CPT	24341	Repair, tendon or muscle, upper arm or elbow, each tendon or muscle, primary or secondary (excludes rotator cuff)	No	Yes	No
rct_cpt_image_include	CPT	0055T	Computer-assisted musculoskeletal surgical navigational orthopedic procedure, with image guidance based on CT/MRI images (list separately in addition to code for primary procedure)	No	Yes	No
RCT_icd10_image_include	ICD10CM	BP3FYZZ	MRI upper extremity left, with contrast	No	Yes	No
RCT_icd10_image_include	ICD10CM	BP3EZZZ	MRI upper extremity of the left	No	Yes	No
rct_cpt_image_include	CPT	76880	[Expired] ultrasound, extremity, nonvascular, real-time with image documentation	No	Yes	No
rct_cpt_image_include	CPT	78662	Ultrasound, limited, joint or other non-vascular extremity structure (i.e., joint space, peri-articular tendon[s], muscle[s], nerve[s], other soft tissue structure[s], or soft tissue mass[es]) real-time with image documentation	No	Yes	No
rct_cpt_image_include	CPT	78661	Ultrasound, complete joint (i.e., joint space and peri-articular soft tissue structures) real-time with image documentation	No	Yes	No

Abbreviations: CPT, current procedural terminology; ICD, international classification of diseases; UTSW, UT Southwestern Medical Center; VUMC, Vanderbilt University Medical Center.

### Data Comparison and Verification Process

**Fig. 1:**
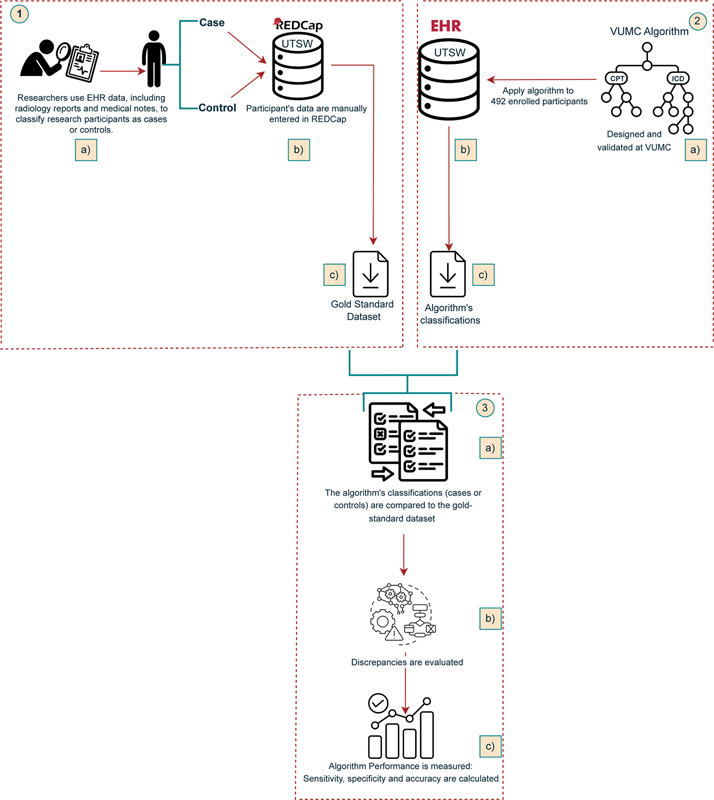
Visual representation of our methodology.

We utilized R (an open-source programming language for data analysis) to compare the algorithm's output classifications (cases or controls) with those in the gold-standard dataset, focusing on identifying discrepancies such as false positives, false negatives, and missing cases between the two sets. To assess the source of these differences, we performed a thorough manual review of each participant's medical chart. This was an essential step to understand how to address the discrepancies and improve the algorithm. Lastly, we calculated the algorithm's sensitivity, specificity, and accuracy. Fig. 1 shows a visual representation of our methodology.

## Results

**Fig. 2:**
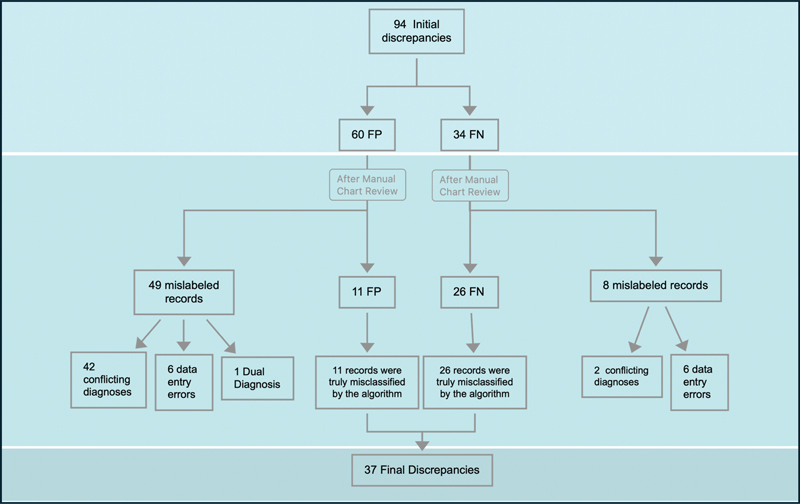
Discrepancies between algorithm outcomes and existing classifications in REDCap. The chart displays 94 discrepancies, categorized into 60 false positives (FP) and 34 false negatives (FN).

**Fig. 3:**
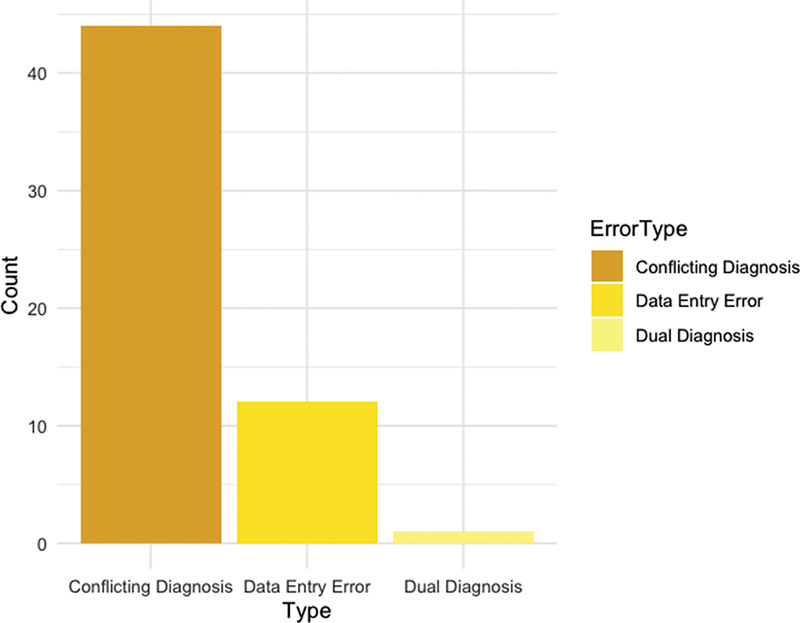
Causes of discrepancy within the algorithm and the gold standard.

Initially, the algorithm identified 398 (80.9%) patients correctly as cases or controls (371 true positive [TP] cases and, 27 true negative [TN] controls). There were 60 false positives (FP), and 34 false negatives (FN). We examined the 94 discrepancies (60 FP and 34 FN) between the algorithm's outcomes and the existing case-control determinations based on the GWAS study in REDCap ( Fig. 2 ). Through a manual review of the medical records, including image impressions, procedures, and clinical notes, we discovered that only 11 of the 60 FP cases (18.3%) were truly false positives. The remaining 49 records (81.7%) were mislabeled in our gold standard database in REDCap. Of these 49 records, 42 (85.7%) had conflicting diagnoses recorded with radiologists identifying an RCT based on imaging, while treating physicians labeled these cases as tendinitis or dyskinesis. Additionally, in six cases research staff made data entry errors. A single patient had two diagnoses, including RCT and GHOA. For the 34 FN cases, we found that only 26 (76.5%) were true misclassifications by the algorithm. The remaining eight records were mislabeled in our gold standard in REDCap, with six being data entry errors and two having conflicting diagnoses where radiologists did not diagnose RCT, but the treating physicians did. Fig. 3 illustrates all discrepancies with the gold standard identified for the false positive and negative cases. Specifically, it shows 44 cases with conflicting diagnoses, 12 data entry errors, and 1 case with a dual diagnosis.

After this thorough review, we reclassified the records and determined that the algorithm produced 420 TP, 26 FN, 11 FP, and 35 TN. Lastly, metrics were recalculated, resulting in a sensitivity of 94%, specificity of 76%, and accuracy of 92%. Ultimately, the true number of discrepancies was 37 (11 FP and 26 FN). Table 4 shows a matrix with our results adjusted for errors in our gold standard.

**Table 4:** Performance metrics

	Actual cases	Actual controls	Performance metric
Labeled as case	420	11	Sensitivity 94%
Labeled as control	26	35	Specificity 76%
			Accuracy 92%

## Discussion

**Fig. 4:**
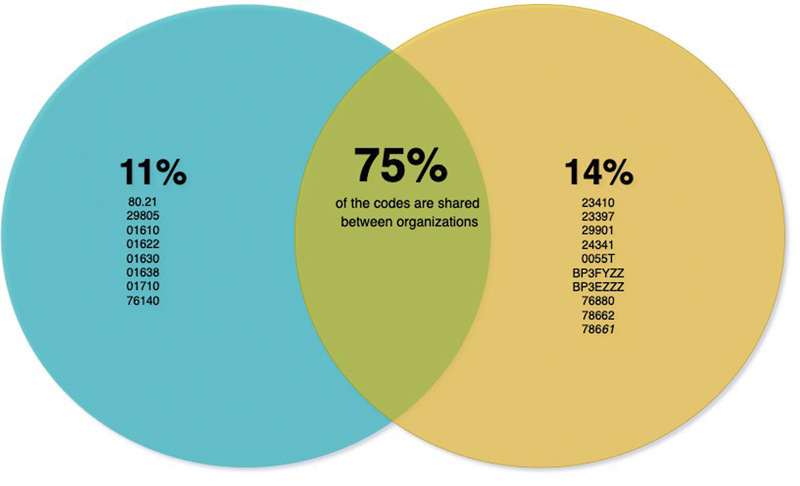
Distribution of shared and unique codes between organizations. The blue section represents the percentage of additional ICD/CPT codes used exclusively at UTSW Medical Center, while the yellow section indicates the percentage of codes unique to the VUMC. The overlap between the blue and yellow areas represents the percentage of codes shared by both organizations.

We implemented an external algorithm that classified cases and controls for an atraumatic RCT study in our EHR and faced several challenges: 1) the initial extraction process failed to identify 33 patients out of the 492 participants due to differences in usage of CPT codes between the organization where the algorithm was originally developed (VUMC) and the organization where the algorithm was applied (UTSW). For example, the procedure for the “repair of the ruptured musculotendinous cuff,” was coded as 23412 in one EHR system and 23410 in the other. These differences extended beyond individual procedures. We observed that some ICD and CPT codes were not included in our initial data dictionary because they were represented by different codes in other institutions. Additionally, we identified the need to account for patients whose imaging studies were performed externally and thus required the inclusion of specific CPT codes associated with these external images. To address these discrepancies, we expanded the algorithm's data dictionary to include additional local CPT and ICD-9 codes that were unique to UTSW Medical Center. Fig. 4 shows the percentage of additional codes that were unique to UTSW Medical Center (11%), the percentage of codes that were unique to VUMC Medical Center (14%), and the percentage of codes that were shared between institutions (75%). Table 3 displays all shared codes between both organizations.

Following our modifications, the algorithm successfully identified most cases and controls, demonstrating the effectiveness of the updated data dictionary and coding practices in harmonizing patient records across different institutional EHR systems. While this reconciliation process was labor-intensive, it provided significant insights into the variability of coding practices between different EHR systems. For example, the identification of locally defined codes as well as a small percentage of procedures coded differently across EHRs highlights the importance of meticulous code verification and standardization in multicenter studies to ensure data integrity and comparability. Metadata sharing prior to data collection for such multicenter studies could emphasize potential coding discrepancies and decrease time-consuming tasks such as manual EHR review.

Additionally, we found 94 discrepancies between the algorithm's outcomes and the existing classifications in our gold standard, which prompted us to perform a thorough manual review of these records during which we found a significant number of mislabeled patients in our gold standard database reducing the true number of discrepancies to only 37 (11 FP and 26 FN). The implementation of the VUMC algorithm allowed us to improve the quality of our gold standard enhancing the accuracy and reliability of patient identification and classification in our institution.

An important aspect to consider is the very definition of the “gold standard” against which algorithms and clinical judgments are compared. The observed discrepancies in our findings largely stem from differences in provider interpretations, particularly between radiologists and other specialists such as orthopedic surgeons and physiatrists. This raises critical questions about the role of disciplinary perspectives in clinical decision-making. Notably, the algorithm appears to align most closely with radiologists' determinations, likely because it is designed around radiology report impressions. This observation highlights the nuanced nature of algorithmic performance, which may be influenced by the specific clinical lens through which evidence is interpreted.

We anticipate that the implementation of the modified algorithm in other performance research sites would likely show further coding discrepancies, but the return would likely be diminished for each additional institution resulting in an algorithm that could be applied in other tertiary medical centers using ICD and CPT codes.

Ensuring data consistency and integrity is paramount for producing valid and reproducible research outcomes. 25 By addressing the diverging coding practices and harmonizing them, we improved the robustness of our dataset, which is essential for drawing meaningful conclusions in clinical studies. Moreover, this implementation highlighted the need for standardized coding systems and meticulous data verification processes, ultimately contributing to the advancement of data interoperability and quality in multicenter research.

### Limitations

One limitation of this study is the inherent variability in coding practices across different medical centers, which impacted the initial performance of the VUMC algorithm when applied to our patient population. Another limitation is that the algorithm was only tested at a single institution, which limits the generalizability of the findings. Testing the algorithm in different organizations could reveal additional coding discrepancies and further affect its performance. This emphasizes the importance of validating such algorithms across diverse settings to ensure their robustness and adaptability in multicenter research studies.

## Conclusion

Implementing and validating the VUMC algorithm at UTSW, an institution with its own patient population and health system, suggests that this tool can perform reliably outside its original development environment. While coding discrepancies need to be addressed, we showed that a rule-based algorithm could be a potential alternative to better identify and validate multi-site patient cohorts. Additionally, the algorithm allowed us to pinpoint 12 data entry errors in our gold standard and gave us an opportunity to validate our classifications.

## Clinical Relevance Statement

The study highlights the critical importance of harmonizing CPT and ICD codes across institutions to ensure accurate patient classification in multicenter studies. Practitioners should be aware that algorithm performance may vary depending on coding practices and the clinical interpretation lens. Addressing coding discrepancies improves data quality, ultimately enhancing the reliability of research outcomes and patient care.

## Multiple-Choice Questions

1. Which challenges were faced during the algorithm implementation for the rotator cuff tear study?
  - Lack of patient consent
  - Differences in CPT code usage across organizations
  - Insufficient sample size
  - Limited imaging availability
  **Correct Answer:** The correct answer is option b. Differences in CPT code usage across organizations.
2. What was identified as a necessary modification to improve the algorithm's performance?
  - Reducing the patient sample size
  - Changing the software used for data analysis
  - Increasing the number of healthcare providers involved
  - Expanding the algorithm's data dictionary to include additional CPT and ICD codes
  **Correct Answer:** The correct answer is option d. Expanding the algorithm's data dictionary to include additional CPT and ICD codes
3. What criteria were used to classify patients as cases in the study?
  - Patients older than 50 years with shoulder pain
  - Patients with a shoulder MRI indicating adhesive capsulitis
  - Patients with a shoulder MRI showing evidence of an atraumatic rotator cuff tear (RCT)
  - Patients with any shoulder-related condition documented in their medical chart
  **Correct Answer:** The correct answer is option c. Patients with a shoulder MRI showing evidence of an atraumatic rotator cuff tear (RCT)
